# Yeast R2TP Interacts with Extended Termini of Client Protein Nop58p

**DOI:** 10.1038/s41598-019-56712-4

**Published:** 2019-12-27

**Authors:** Ge Yu, Yu Zhao, Shaoxiong Tian, Jay Rai, Huan He, John Spear, Duncan Sousa, Jinbo Fan, Hong-Guo Yu, Scott M. Stagg, Hong Li

**Affiliations:** 1 Department of Chemistry and Biochemistry, Florida State University, Tallahassee, FL 32306 USA; 2 Institute of Molecular Biophysics, Florida State University, Tallahassee, FL 32306 USA; 3 Department of Biological Science, Florida State University, Tallahassee, FL 32306 USA

**Keywords:** Proteins, RNA, Structural biology, Biochemistry, Biophysics, Structural biology

## Abstract

The AAA + ATPase R2TP complex facilitates assembly of a number of ribonucleoprotein particles (RNPs). Although the architecture of R2TP is known, its molecular basis for acting upon multiple RNPs remains unknown. In yeast, the core subunit of the box C/D small nucleolar RNPs, Nop58p, is the target for R2TP function. In the recently observed U3 box C/D snoRNP as part of the 90 S small subunit processome, the unfolded regions of Nop58p are observed to form extensive interactions, suggesting a possible role of R2TP in stabilizing the unfolded region of Nop58p prior to its assembly. Here, we analyze the interaction between R2TP and a Maltose Binding Protein (MBP)-fused Nop58p by biophysical and yeast genetics methods. We present evidence that R2TP interacts largely with the unfolded termini of Nop58p. Our results suggest a general mechanism for R2TP to impart specificity by recognizing unfolded regions in its clients.

## Introduction

Box C/D type of small nucleolar ribonucleoprotein (snoRNPs) particles are essential ribosome biogenesis factors that function in methylation and processing of ribosomal RNA (rRNA)^1–7^. A large majority of box C/D snoRNPs consists of four proteins and one box C/D snoRNA but some can assemble into large RNPs such as the small subunit (SSU) processome^8,9^. The minimally assembled box C/D snoRNPs act as site-specific methyltransferases and they contain, in yeast, Nop1p (or fibrillarin), Nop56p, Nop58p, Snu13p, and a box C/D snoRNA. The SSU contains the same five components plus additional assembly factors, ribosomal subunit proteins, and unprocessed rRNA^9,10^. The megadalton SSU processes rRNA co-transcriptionally into the transcripts required for the two ribosomal subunits, in which the U3 box C/D snoRNP module plays multiple roles in assembly and conformational transitions^11–13^.

Biochemical and structural characterization indicate that box C/D snoRNPs do not assemble properly for function *in vitro* whereas their archaeal homologs do^14^. The eukaryotic box C/D proteins have high sequence homology to their archaeal counterparts but often contain charged extensions that could prevent spontaneous folding or assembly. As a result, eukaryotic cells benefit from chaperone molecules in assisting assembly and maturation of box C/D snoRNPs^15,16^. The yeast R2TP (Rvb1p, Rvb2p, Tah1p, YCR060W and Pih1p, YHR034C) complex was previously identified to be responsible for maturation of the box C/D snoRNP^17–20^. In mouse cells, Rvb1/2 were co-purified with polyA-tagged U14 snoRNP from nuclear extract^21^ and later shown to be required for snoRNP stability and nucleolus localization^22^. In yeast, Pih1p interacts both genetically and physically with the box C/D snoRNP proteins Nop58p and Snu13p^23–27^. Furthermore, depletion of Pih1p led to accumulation of rRNA intermediate and reduction of polysomes^23^. Despite the convincing roles of R2TP in box C/D snoRNP maturation, detailed molecular mechanism of box C/D snoRNP maturation remains elusive.

**Figure 1:**
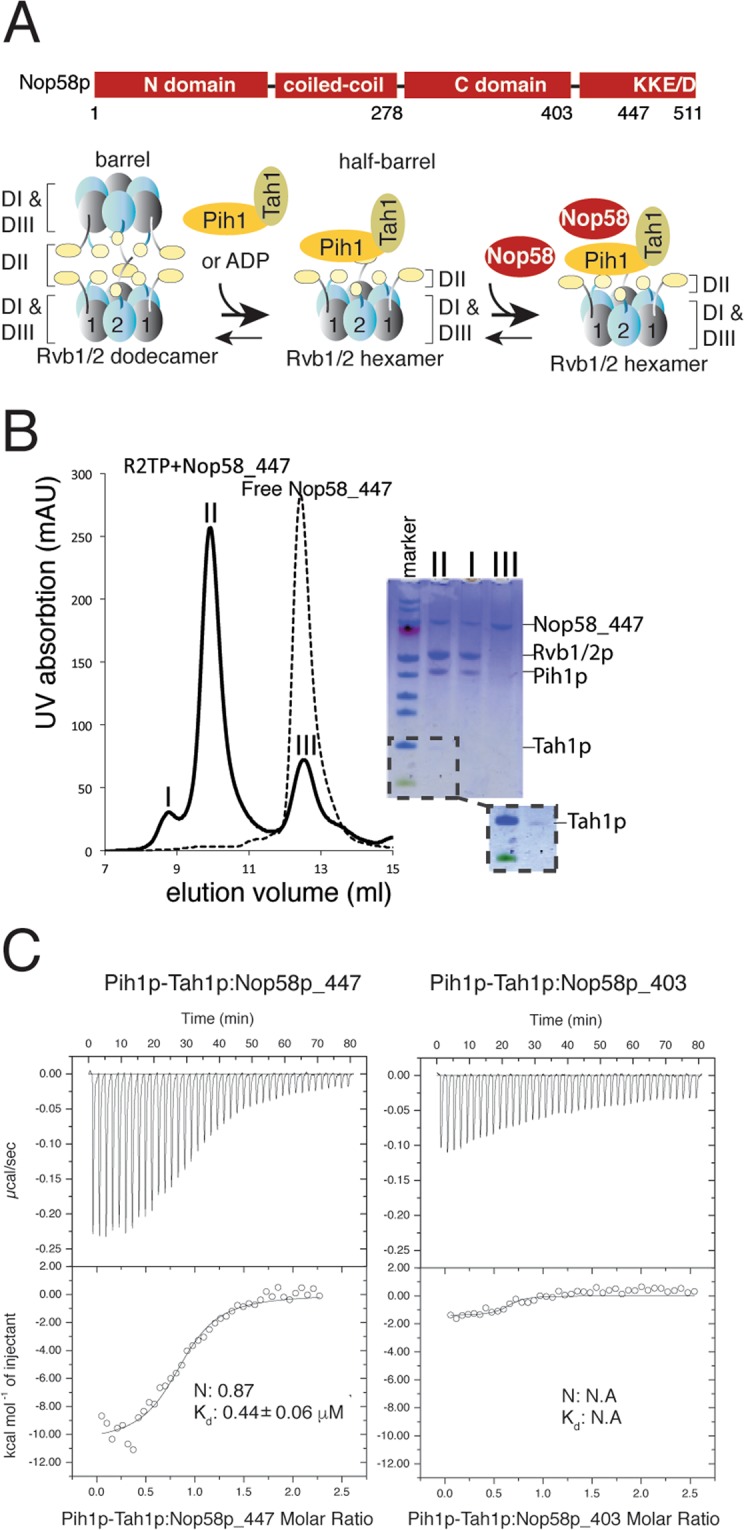
Nop58p features, purification and characterization of R2TP-Nop58p. (**A**) Primary structural features of Nop58p and its schematic binding model to R2TP (based on Tian *et al*. 2017). Key regions are labeled as those used in this article. Rvb1/2p are depicted as oval objects with protrusions representing the flexible domain DII. Binding of Pih1p-Tah1p leads to dissociation of Rvb1/2p dodecamer (barrel) to heterohexamers (half barrel). Nop58p binds to the half barrel. (**B**) Reconstitution of R2TP and its complex with MBP-Nop58p_447 (1–447). Individually prepared MBP-Nop58p_447 and R2TP were mixed and first loaded to an amylose affinity column followed by a gel filtration column. Elution peaks of the gel filtration column were analyzed on a SDS-PAGE gel and used for subsequent studies. (**C**) Stoichiometry and binding constant of MBP-Nop58p_447 and MBP-Nop58p_403 for Pih1p-Tah1p heterodimer measured by Isothermal Titration Calorimetry (ITC). The fitted molar ratio (N) and binding constant (K_d_) for MBP-Nop58p_447 are included in the isotherm plot and those for MBP-Nop58p_403 were not obtained due to weak binding.

Two recent electron cryomicroscopy (cryoEM) studies revealed a consistent structural model of R2TP^28,29^. The two AAA + (**A**TPase associated diverse cellular **a**ctivities) ATPases, Rvb1p, and Rvb2p, assemble into a heterohexamer that forms a circular base onto which one or two heterodimers of Pih1p and Tah1p dock (Fig. 1A). The Rvb1/2p base contains two layers: the bottom layer is made of the core AAA + module (domains I and III, or DI & DIII) that includes Walker A and B motifs, sensor domains 1 & 2, and the arginine finger; the top layer is made of the Rvb1/2-specific domain, or Domain II (DII). The intrinsically disordered regions (IDRs) of Pih1p, flanked by its N-terminal PIH domain (34–166) and the C-terminal domain (268–344), provide the primary contacts to DII of Rvb1/2p^28,29^. Tah1p plays a less role in contacting Rvb1/2p but is believed to mediate binding of the molecular chaperone heat **s**hock protein 90 (Hsp90) through its N-terminal TPR (the Tetratricopeptide repeats) motifs^27,30,31^. In these studies, although the hexameric Ruv1/2p ring can be resolved at an atomic resolution, structural characterization of the other components has been difficult owing to their flexibility^28,29^. The exact stoichiometry of Pih1p-Tah1p and how they are oriented with respect to the symmetric ATPase ring remain completely unknown. Recently, study of human R2TP complex has made significant progress, although it also faces the same challenge in characterizing the PIH1D1-RPAP3 (Pih1p-Tah1p homolog) structure^32,33^. Nonetheless, several aspects of the human R2TP structure provide a basis for examining yeast R2TP structural models. In human R2TP, the N-terminal portion of RPAP3 (equivalent to Tah1p) protrudes from the RUVBL1/2 ring without interacting with the ATPases^33^. Deletion of the mobile N-terminal portion of RPAP3 enabled placement of PIH1D1 as a single copy on top of the ATPase ring with its PIH domain bound directly to one RUVBL2^32^. Given the high homology between yeast and human R2TP, the new human R2TP structures provide a basis for examining how R2TP binds its client protein Nop58p.

Previous studies of Nop58p-R2TP interactions have been limited at the level of genetics and biochemistry. These studies have all identified the C-terminus of Nop58p as a likely site for binding Pih1p of R2TP. Gonzales *et al*. and Prieto *et al*. showed by yeast two-hybrid that Nop58p 217–512 or 324–512 interacts with Pih1p^23^. Through co-purification, Quinternet *et al*. showed that Pih1p interacts with Nop58p 159–412^27^ while Kakihara *et al*. showed that Pih1p interacts with Nop58p 285–345^25^. We previously performed cross-linking and analytic ultracentrifugation studies by mixing a truncated Nop58p, Nop58p_447, with R2TP, that also identified C-terminus of Nop58p as a site for binding R2TP^28^. However, there has not been an attempt in visualizing R2TP when bound to Nop58p. Here, we reconstituted a Nop58p_447-bound R2TP complex (Fig. 1B) and characterize its architecture by cryoEM, protein-protein cross-linking, and yeast genetics. In light of the previously known client free R2TP structures both from human and yeast, we are able to place Nop58p at the top of the R2TP complex in a manner consistent with all experimental data.

## Results

### Thermodynamic evidence for Pih1p interaction with The C-terminus of Nop58p

We expressed and purified a truncated version of Nop58p, Nop58p_447 (lacking residues 448–511) from *S. cerevisiae* fused with the Maltose Binding Protein (MBP) at its N-terminus (MBP-Nop58p_447), for stability reasons. Previous studies showed that this construct did not disrupt the function of box C/D snoRNP^34^. To reconstitute the MBP-Nop58p_447-R2TP complex, we prepared free MBP-Nop58p_447 by amylose affinity and size-exclusion chromatography and then incubated with previously reconstituted R2TP complex. We found that the incubated MBP-Nop58p_447 specifically bound with R2TP on a size-exclusion column, which allowed isolation of the MBP-Nop58p_447-R2TP complex (Fig. 1B). To better understand the chemical nature of MBP-Nop58p_447 binding to R2TP, the purified MBP-Nop58p_447 was first used in an isothermal titration calorimetry study with Pih1p-Tah1p, which indicates a 1:1 binding stoichiometry and a moderate binding affinity (Fig. 1C). This result, when combined with the previously determined 1:1 stoichiometry of Pih1p-Tah1p to the Rvb1/2p hexamer^28^, suggests a 1:1 stoichiometry of MBP-Nop58p_447 to R2TP. To identify the impact of the C-terminal tail of Nop58p on its interaction with Pih1p-Tah1p, we created Nop58p_403 (lacking residues 404–511) and examined its interaction with Pih1p-Tah1p by ITC studies. Nop58p_403 generated insignificant heat during titration (Fig. 1C), suggesting a significantly reduced binding affinity for Pih1p-Tah1p as compared to Nop58p_447. Thus the region 404–447 plays an important role for Nop58p to bind Pih1p-Tah1p. This region has no defined secondary structure and is only stabilized in the full small subunit processome as an extended peptide (PDBid: 5WLC).

### Evidence for interactions between Nop58_447 termini and R2TP by Cross-linking

**Figure 2:**
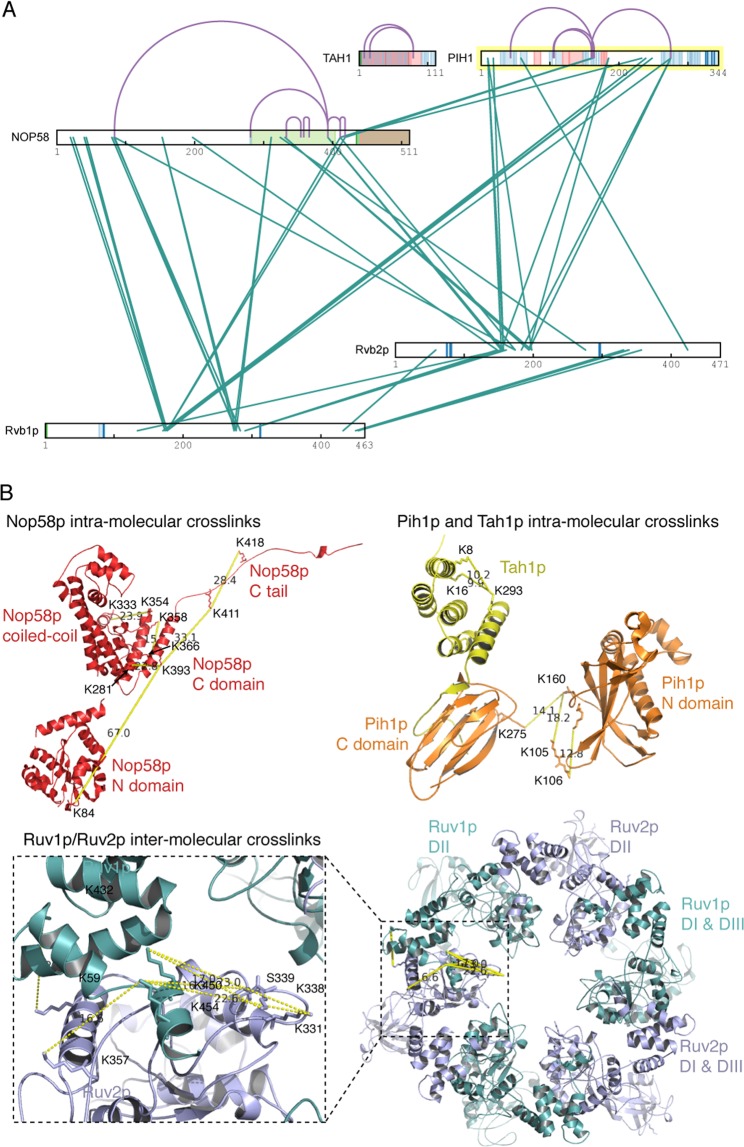
Protein cross-linking results. (**A**) Cross-link map of R2TP in complex with MBP-Nop58p_447. Observed intra-molecular links are colored purple and inter-molecular links are colored teal. Regions are colored according to designations in UniProt Knowledgebase (UniProtKB). (**B**) Three-dimensional map of intra- and inter-molecular cross-links. The cross-links can be found in Table S2. The PDB coordinates of the known structures depicted are 5WLC for Nop58p, 4CGU for Pih1p C domain-Tah1p complex, and 4CHH for Pih1p N domain. The Rvb1/2p complex coordinates were obtained by homology modeling.

The previous cross-linking study between Nop58p_447 and R2TP was performed on R2TP incubated with Nop58p_447^28^. To ensure the cross-links are obtained from fully assembled R2TP-Nop58p_447 complex, we performed lysine-specific chemical cross-linking followed by mass spectrometric identification^35^ similarly as that carried out previously^28^. The cross-links were consistent with but more extensive than those obtained from the incubated samples^28^. We first analyzed intra-molecular cross-links for Pih1p, Tah1p, and Nop58p_447 and compared to the distances obtained from the available crystal (Pih1p and Tah1p) or cryoEM (Nop58p) structures of the individual proteins (Table S1). The intra-molecular cross-links largely fit well to the distances of the lysine residues within individual domains (Fig. 2A,B) with one exception for Nop58p_447. The cross-link between Lys84 of the N domain and Lys393 of the C domain of Nop58p (Fig. 2B) is not consistent with the Nop58p structure (PDBid: 5WLC) or any of the known archaeal Nop5 structures^36^. In these known Nop protein structures, the N and the C domains of folded Nop5 or Nop58p are too far apart to accommodate the observed lysine-lysine cross-links. This result indicates that the N domain of Nop58p bound with R2TP may have a different conformation with respect to the C domain. Additionally, since there is no crystal structure of the full-length Pih1p, the intra-molecular cross-link between Lys275 in its C domain and Lys160 in its N domain cannot be confirmed (Fig. 2B). Nonetheless, the overwhelming agreement between intro-molecular cross-links and crystal structure-derived distances validates the cross-linking method.

The inter-molecular cross-links between Rvb1p and Rvb2p were then examined and compared to the Rvb1p/Rvb2p structure model obtained previously^28^. Inter-molecular cross-links within domains DI and DIII fit very well to the structure model (Fig. 2B), again validating the accuracy of the cross-linking method. The cross-links between the flexible DII of the two proteins can only fit to the Rvb1/2p dodecamer model that is believed to be present at a small fraction in the cross-linking sample (Fig. 2B).

When examining inter-molecular cross-links, extensive cross-links between Nop58p_447 and R2TP, especially DII of Rvb1/2p and Pih1p, were observed and mapped to the interior of the R2TP barrel (Fig. 2A and Table S2). In general, the N domain of Nop58p_447 interacts mostly with Rvb1p and its C domain with Rvb2p (Fig. 2A). Nop58p_447 forms more extensive interactions with Rvb1p than with Rvb2p as indicated by its contacts with residues in DII_INT_ of Rvb1p (Lys274, Lys277 and Lys283). These residues are located more interior of the hexameric ring. Nop58p_447 also interacts with residues in DII_EXT_ of both Rvb1p (Lys174 and Lys177) and Rvb2p (Ser148, Lys154, Lys 157, Lys174, Lys194 and Lys198). Lastly, the IDR and residues located close to IDR of Pih1p are cross-linked to the C terminal tail of Nop58p_447 (Lys411 and Lys418) (Fig. 2A). These results suggest a placement of Nop58p_447 consistent with it being on top of R2TP hexamer.

### Overall structure of Nop58_447 interaction with R2TP

**Figure 3:**
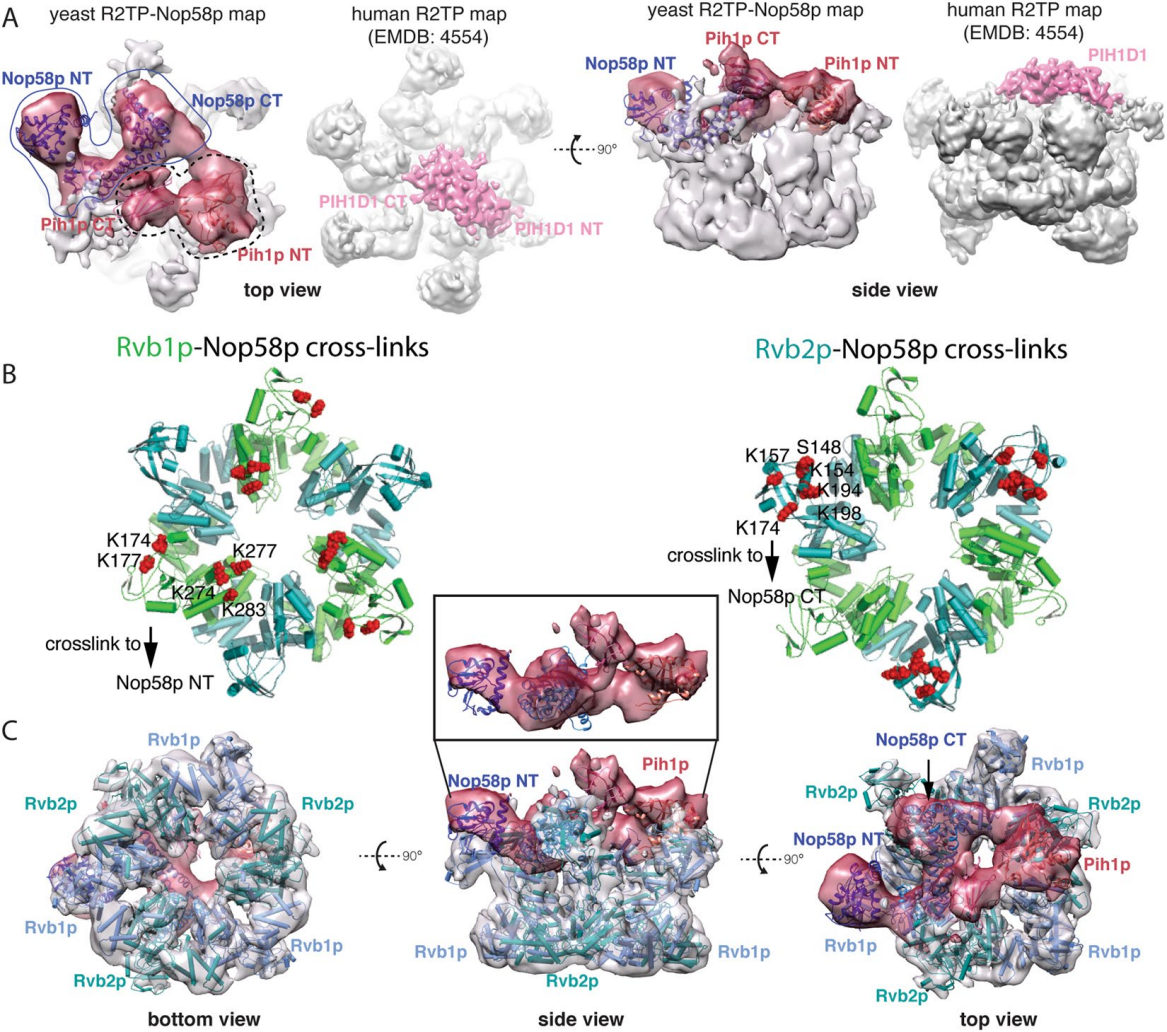
CryoEM structure of R2TP in complex with MBP-Nop58p_447. (**A**) Two orthogonal views of the composite electron density of the MBP-Nop58p_447 bound with R2TP (R2TP-Nop58p) in comparison with that of human R2TP (EMDB 4554). The density assigned to Rvb1/2p is from C3-symmetry refined and colored in gray. The density assigned to Pih1p and Nop58p is from C1-refined and colored in red. The density from human R2TP lacking the N-terminal domain of RPAP3 (R2TP-DNT, EMDB: 4554) is first aligned with that of R2TP-Nop58p_447 and shown next to it. The PIH1D1 identified by ref. ^32^ is colored in pink. Yeast Pih1p is assigned similarly as PIH1D1 from ref. ^32^ and shown by the fitted crystal structures of Pih1p N- and C-terminal domains (Pih1p NT and Pih1p CT). The remaining top density is assigned to Nop58p with one possible placement indicated by the yeast Nop58p structural model (PDBid: 5WLC). (**B**) Mapping residues of Rvb1p and Rvb2p that are cross-linked to Nop58p to structure models of the Rvb1/2p complex. Left, residues of Rvb1p (green) are shown in red spheres and are mostly crosslinked to Nop58p N-terminal domain (Nop58p NT). Right, residues of Rvb2p (teal) are shown in red-spheres and are mostly crosslinked to Nop58p C-terminal domain (Nop58p CT). (**C**) The composite density of the MBP-Nop58p_447 bound with R2TP (R2TP-Nop58p) as shown in Panel A is shown with a Rvb1/2p model fitted. The Rvb1/2p density is transparent and colored in gray with the fitted Rvb1/2p in cartoon representation. The density identified as Pih1p and Nop58p is colored in red and fitted with the available partial structures of Pih1p and Nop58p. Inset shows isolated density assigned to Nop58p and Pih1p.

We next carried out cryoEM analysis of the reconstituted R2TP_MBP-Nop58_447 complex (Fig. 3). Two-dimensional classification of ice-embedded specimens displayed averages with both disc-like end-on and rice bowl-shaped side views, similar to those observed for R2TP^28^ and SWR-C^37^ (Figs. S1 and S2). Following extensive sorting by removing particles that are either incomplete or overly populated top-views, we arrived at 179,483 particles from which we refined the model by applying the C1 and C3 symmetries, respectively. The C3-refined model took advantage of the three-fold symmetry in the Rvb1/2p heterohexamer and yielded an overall resolution of 6.3 Å based on the gold standard FCS_0.143_ method (Fig. S3). The C1-refined model reached an overall resolution of 8.9 Å (FCS_0.143_) (Fig. S3), clearly revealing the segments corresponding to the Rvb1p/Rvb2p base and a top likely belonging to Pih1p-Tah1p and MPB-Nop58p_447 (Fig. 3A). Local resolution analysis^38^ is consistent with the overall estimate with the base region at 7–8 Å and the central region at 9–11 Å resolution for the C1-refined model (Fig. 3B). To help overcome the conformational heterogeneity, we performed multi-body refinement^39^, which confirmed the heterogeneity at the top but did not further improve the resolution.

We used the C3-refined reconstruction to analyze Rvb1/2p structure and the C1-refined reconstruction to analyze that of Pih1p-Tah1p and MPB-Nop58p_447 (Fig. 3C). The Rvb1/2p structure model can be unambiguously placed into the base of the 6.3 Å map, which allowed distinction between Rvb1p and Rvb2p based on the different length of the last helix as well as the orientation of the domain II (DII) of both subunits (Fig. S4). Similar to previously determined R2TP structures, DII of both Rvb1/2p is rotated slightly from that in the Ct. Rvb1/2p structure, consistent with the binding of Pih1p-Tah1p and Nop58p_447. The density for Rvb1/2p base in the C1 refined map is also similar to that C3 refined except that DII of Rvb1p exhibits more asymmetrical features, likely reflecting asymmetrical binding of Pih1p-Tah1p and MBP-Nop58p_447 (Fig. 3C).

The density above Rvb1/2p in the C1-refined reconstruction, especially following multi-body refinement (Fig. 3A & Fig. S5), contains a segment similar to that assigned to PIH1D1 in the 4.0 Å human R2TP structure^32^. Similar to human PIH1D1, it is connected to one RUVBL2 subunit whose DII is distorted, and its size accommodates both the N-terminal PIH and the C-terminal CT domain (Fig. 3A). Placement of Pih1p at this location lead to an excellent agreement with our cross-linking data where the PIH domain primarily interacts with DII of Ruv2p and the IDR interacts with the adjacent Ruv1p (Table S2). Similar to human R2TP where RPAP3 (Tah1p homolog) is flexible and in agreement with its lack of cross-links to Ruv1/2p (Table S2), Tah1p is likely not part of the central density. As there is complete lack of cross-links for MBP (Table S2), it also unlikely contributes to the central density. However, to evaluate if the MBP tag fused to Nop58p_447 has an impact on the observed structure, we purified the R2TP complex bound with a tagless Nop58p_447 (R2TP-Nop58p_447) and performed single particle reconstruction of this complex to an overall resolution of 10.7 Å (Figs. S4–S6). Comparison of its electron density to that of R2TP bound with MBP-Nop58p_447 indicates a general agreement between the two structures but re-arrangement in top density is observed (Fig. S5). This result supports the absence of MBP from the central density.

The remaining density after placing Pih1p is thus assigned to Nop58p_447. Rigid docking of a known structure of Nop58p (PDBid: 5WLC) or its homolog did not allow a satisfactory fit to the density. However, it is likely that isolated Nop58p free from bound RNA or fibrillarin adopts a different conformation. Evidence for the alternative conformation is found in the observed cross-link between the N-terminal and C-terminal domains (Table S1) that is not expected in all currently known Nop58p. Flexibility of the Nop58p family of proteins is well documented^40^. Thus, even though a precise placement of Nop58p_447 is not possible, a binding model based on cross-links and the shape of the density is suggested in Fig. 3A. Nop58p_447 primarily uses its N domain to cross-link Rvb1p DII and C domain to cross-link Rvb2p DII. The C terminus further interacts with Rvb2p and Pih1p simultaneously and is thus placed near Pih1p and Rvb2p (Fig. 3A,C) & Table S2). This structure model accommodates the biochemical and cross-linking data.

### Genetic evidence for interactions between Nop58p C terminus and Pih1p

**Figure 4:**
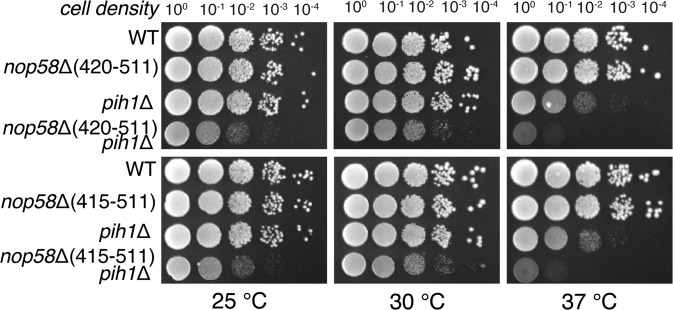
Yeast double depletion results. Yeast strains were grown to saturation, 10-fold serially diluted, spotted onto YPD and incubated at 25 °C, 30 °C and 37 °C, respectively for each of the indicated strains.

In order to test the functional importance of the observed Nop58p interactions with R2TP, specifically with Pih1p, we created double depletion yeast strains and tested the impact of the double deletions on growth. We first created yeast strains that lack Pih1p (*pih1*Δ)or Nop58 C-terminus (*nop58*-Δ(415–511) and *nop58*-420Δ(420–511)), respectively. We then created double mutants that lack both Pih1p and Nop58 C-terminus (*pih1*Δ/*nop58*-Δ(415–511) and *pih1*Δ/*nop58*-Δ(420–511)) by sporulation of the heterozygous diploids and dissection of the tetrads. As shown in Fig. 4, individually depleted yeast strains showed little (*nop58*-Δ(415–511) to moderate (*pih1*Δ) growth defects at the elevated temperature of 37 °C. Both strains had comparable growth rate as the wild-type at 30 °C. In contrast, both the double mutants, *pih1*-Δ/*nop58*-Δ(415−511) and *pih1*-Δ/*nop58*-Δ(420−511), showed severe defects in growth at all temperatures (Fig. 4), suggesting a strong genetic interaction between Pih1p and Nop58p C terminus consistent with the cryoEM and cross-linking results.

## Discussion

The R2TP complex is a box C/D snoRNP assembly factor that impacts snoRNP stability and nucleolar localization through its interaction with Nop58p^17,18,20,22–24,27,41,42^. We show for the first time that Nop58p interacts with both Rvb1/2p and Pih1p in assembled R2TP through its unfolded termini. Our finding recapitulates the previously established hexameric R2TP as the functional chaperone model and the role of R2TP in stabilizing nearly folded client proteins.

The hexameric R2TP model has important implications in R2TP specificity. Rvb1p and Rvb2p belong to the AAA + ATPase family and are associated with multiple chaperone processes, likely owing to its ability to deliver nucleotide-driven movement and its broad binding specificity^19^. Adaptor proteins help to direct Rvb1/2p to specific client molecules. It had been shown previously that Pih1p-Tah1p is capable of switching the oligomeric state of Rvb1/2p from dodecamer to hexamer^28,29^. Other adaptor proteins such as Ino80 insertion domain were shown to stabilize the dodecamer form of Rvb1/2p^43^. Our finding that Nop58p binds the hexameric R2TP suggests that Pih1p and Tah1p are specific adaptors of Rvb1/2p for Nop58p. Similarly, other adaptors can switch Rvb1/2p to dodecamer for specific clients to bind. The fact that Nop58p also interacts with Pih1p further aids the specific chaperone activity of R2TP on snoRNP maturation.

We showed that the unfolded extensions of Nop58p play important roles in its interaction with R2TP. Notably, the C-terminal extension (403–437) is captured in the cryoEM structure of the 90 S pre-ribosome where it is fully extended and completely embedded by surrounding assembly factors^44^. Thus, defects in maturation of this extension could lead to mis-assembly of the 90 S pre-ribosome. Importantly, the sequence beyond 447 contains 13 repeats of the charged peptide motif KKE/D that may further require the chaperone function of R2TP. The fact that R2TP interacts with the unassembled Nop58p at this region suggests an important role of R2TP in 90 S pre-ribosome assembly. This is consistent with the experimental data that depletion of R2TP leads to accumulation of the U3 snoRNA and unprocessed rRNA^23,26^.

## Methods

### Protein expression and purification

Cloning and purification of R2TP and Nop58p_447 were described previously^28^. To express the Maltose Binding Protein (MBP)-fused Nop58p_447, the gene expressing residues 1–447 of Nop58p fused with an N-terminal MBP tag was cloned into the pMal-c2x vector to yield pQMBPNop58p_447. Separately purified His-Pih1p-Tah1p and Rvb1/2p heterocomplexes were incubated and co-purified on a Ni-NTA affinity column. The eluted complex was treated with TEV protease to remove the his-tag and isolated by size exclusion chromatography. MBP-Nop58p_447 was purified from *E. coli* cells transformed with pQMBPNop58p_447. Cells were grown at 37 °C until OD600 reached 0.6–0.8 before being induced with 0.2 mM isopropyl β-D-1-thiogalactopyranoside (IPTG). The cells were allowed to grow at 16 °C overnight before being harvested at 4 °C by pelleting at 6000 × g for 15 min in a Beckman Coulter Avanti J-20 XPI centrifuge (OPTIMA). The cell pellets were suspended in buffer A (25 mM sodium phosphate, pH 7.5 400 mM NaCl, and 5% glycerol) and lysed by sonication. The clear supernatant was then applied to an Amylose column equilibrated with buffer A. The bound sample was washed with buffer B (25 mM sodium phosphate, pH 7.5, 400 mM NaCl, and 5% glycerol) and eluted with buffer B supplemented with 10 mM maltose. The eluted MBP-Nop58p_447 was further purified by ion-exchange chromatography with a monoQ (GE Health Sciences) and gel filtration chromatography with a Superdex Increase 200 10/300 GL column (GE Health Sciences). To purify the MBP-Nop58p-bound R2TP, MBP-Nop58p_447 was incubated overnight at 4 °C with previously purified R2TP and applied to an Amylose column and the eluted factions were further purified on a gel filtration column in a storage buffer (25 mM sodium phosphate, pH 7.5, 400 mM NaCl, and 5% glycerol). The complex was identified by SDS-PAGE analysis of the fractions (Fig. 1). We found that the R2TP-MBP-Nop58p_447 has better stability and homogeneity than the R2TP-Nop58p_447.

### Isothermal titration calorimetry

Pih1p-Tah1p and MBP-Nop58p_447 samples were exchanged into the binding buffer (25 mM HEPES 150 mM NaCl 5% Glycerol 5 mM beta-ME pH 7.5) on a size exclusion column (GE superdex 200 increase 10/300 GL). Prior to titration, both samples were centrifuged and degassed under vacuum for 10 minutes. MBP-Nop58p_447 was placed into the sample cell at 7.8 μM and Pih1p-Tah1p was loaded into the injector at 99.6 μM concentration. 40 × 6 μl-injections were performed at a constant stirring speed of 310 rpm, with a 120-second spacing at 25 °C. MBP-Nop58p_403 exibited much less heat even at?? concentration (Fig. 1C). The isotherm was fitted using the MicroCal Anaysis software to a One Site model, yielding the stoichiometry value N and binding constant *K*_*d*_ (Fig. 1C).

### Electron microscopy sample preparation

The sample was exchanged to the storage buffer without glycerol for cryoEM studies. The initial sample quality was assessed via negative staining. For negative staining, the continuous carbon copper grids (Electron Microscopy Science) were glow discharged in a Gatan Solarus 950 Plasma cleaner for 20 seconds. 4 μL of the sample was applied to the glow discharged grid and stained with 2% (w/v) uranyl acetate. The ice-embedded specimens were prepared by placing 3 μL of 0.5 mg/ml each complex onto a glow-discharged Quantifoil grids (R 2/2 Jena, Germany). The grids were blotted for 3 seconds at 4 °C in 100% humidity before being plunged into liquid ethane by the use of an FEI Vitrobot MK IV (Hillsboro OR).

### EM data collection and processing

The ice-embedded MBP-Nop58p_447-R2TP samples were imaged with an FEI Titan Krios operated at 300 keV equipped with a DE-64 direct electron camera (Direct Electron, San Diego, CA, USA) in an integration mode. The Leginon software for automatic data acquisition was used^45^. A total of 1,727 images were collected in two sessions at a nominal magnification of 37,000 × with a pixel size of 1.01 Å/pixel and −1.0 to −3.0 microns defocus at the specimen level. A total doze of 61.3 e^−^/Å^2^ was applied over 48 frames at a total exposure time of 1.5 s. The initial stage of image processing were carried out with the Appion pipeline^46^. Frames were aligned and dose compensated by using the alignment script provided by DE as implemented in Appion^47^. Contrast transfer function (CTF) parameters for individual images were estimated with the Automated CTF Estimation (ACE)^48^ followed by CTFFIND4^49^. Particles were picked manually to create templates which were subsequently used for template-based picking using FindEM^50^. In total, 951,048 particles were picked.

2D classification with ROME^51^ removed particles with ice or those of bad qualities resulting in a stack of 683,151 particles. The stack was further classified with RELION 2.0^52^ into five classes using 60 Å low pass filtered R2TP structure (EMDB 8839) as the initial model. Among five classes, one class with 179,483 particles look good and further refined (Fig. S2) with either C1 or C3 symmetry. The C3-refined structure yielded the best map for the circular base of the assembly with an overall resolution of 6.3 Å (with mask) (FSC_0.143_) (Fig. S3) with distorted top. To produce density for top of the assembly, we refined particles with the C1 symmetry, reaching an overall resolution of 8.9 Å (with mask) (FSC_0.143_) (Fig. S3). Additional refinement with the recently implemented Multi-body refinement in RELION-3.0 by defining three bodies (bottom, middle, and top) revealed a substantial movement within the complex and improved the map quality slightly^39^. A composite density consisting of that for Rvb1/2p from the C3-refined and that for Pih1-Tah1-MBP-Nop58_447 from the C1-focused refinement is segmented by use of the watershed segmentation feature available in UCSF Chimera^53^ and shown in Fig. 3. We constructed several atomic model structures of the yeast Rvb1/2 heterohexamer by homology building based on the crystal structure of the *Chaetomium thermophilum* (Ct) Rvb1/2 heterohexamer (PDB code: 4WVY) and human Rvb1 (PDB code: 2C9O). We placed the resulting models of ScRvb1/2 hexamer into the composite map, manually adjusted it COOT^54^ and subjected them to the density-guided refinement procedure implemented in Rosetta^55^. The crystal structures of Pih1p C domain-Tah1p complex (PDBid: 4CGU), Pih1p N domain (PDBid: 4CHH), and the cryoEM structure of Nop58 (PDBid: 5WLC) were placed into the top density guided by the observed inter-molecular cross-links (Table S2). No manual adjustment was made for Pih1p and Tah1p but the orientations of the Nop58p N-terminal domain was adjusted relative to the other domains in order to fit the density. No refinement was carried out for Pih1p, Tah1p, or Nop58p.

The vitrified R2TP-Nop58p_447 specimens were imaged similarly but on a DE20 direct detector (DE, San Diego, CA). A total of 2911 images were collected in movie mode at a nominal magnification of 29,000 × with 1.26 Å/pixel and a defocus range of −1.0 to −3.0 microns. A 103.9 e^−^/Å^2^ dose was applied over 48 frames at a total exposure time of 1.7 s. 3D classification was used to remove junk particles from the initial 399,991 to the final 165,420 particles. Further classification with RELION-2.0 into five classes yielded a best class comprised of 39,360 particles that was subsequently refined to an apparent resolution of 11 Å (FSC_0.143_) (Fig. S6).

### Lysine-specific cross-linking coupled with mass spectrometry

The cross-linking reaction and mass spectrometry were performed similarly as described in ref. ^28^ with exception for the sample used. Briefly, the co-purified MBP-Nop58p_447-R2TP complex analyzed by cryoEM was incubated with 0.5% deuterated (d4) or nondeuterated (d0) bis(sulfosuccinimidyl)suberate (BS3) for 30 min. The BS3-d0 and BS3-d4 cross-linked samples were mixed in equal volume, purified on a 7.5% SDS-PAGE gel, and digested by trypsin for mass spectrometry. Digested peptides were separated on an Easy Nano LC II system (Thermo Fisher Scientific), ionized by electrospray ionization (ESI) and detected by a Velos LTQ-Orbitrap Mass Spectrometer (Thermo Scientific). Cross-linked peptides showed the presence of both BS3-d0 and BS3-d4 cross-linkers, ion pairs separated by 4.0247 Da and were verified mannually.

### Yeast Strain Creation and Spot Dilution Assays

The *pih1*Δ, *nop58*-Δ(451–511) and *nop58*-Δ(420–511) (Nop58p C-terminal truncations) haploid strains were generated using a standard PCR-based gene deletion strategy^56^. To create the *pih1*Δ strain, the entire ORF of *PIH1* was replaced with an HphMX cassette amplified from the plasmid pFA6a-6xGLY-Strep-tagII-hphMX4. The PCR product was transformed into the wild-type *Saccharomyces cerevisiae* BY4742 cells. The transformants were selected on YPD solid medium containing Hygromycin B. Correct replaced strains were confirmed by PCR and DNA sequencing of the *pih1* ORF. Both *nop58*–415Δ and *nop58*–420Δ strains were created by replacing the Nop58p C-terminus coding sequences with a premature stop codon within a KanMX cassette that was amplified from the plasmid pFA6a-3HA-KanMX4^56^. Each PCR product was transformed into the wild-type *Saccharomyces cerevisiae* strain BY4741 cells and the transformants were selected on YPD solid medium containing G418. Correctly replaced C-terminus strains were confirmed by PCR and DNA sequencing of the *NOP58* ORF.

To generate the double deletion strains, heterozygous strains were created by crossing the BY4742 *pih1Δ* haploid strain with the BY4741 *nop58-Δ(415–511)* or the *nop58-Δ(420–511)* haploid strain, which were later sporulated using a liquid sporulation method^57^. At least 20 tetrads were dissected on YPD agar plates from each sporulated heterozygous strain. Tetrad dissection plates were replica plated onto YPD + G418, YPD + Hygromycin B plates for genotyping. Mating types of haploids were determined by crossing with tester strains. Appropriate haploids were then confirmed by PCR and DNA sequencing.

Both the wild-type and deletion strains were grown in liquid YPD medium to saturation followed by two washes and dilution to OD_600_ = 1.0 with sterile ddH_2_O. Cells were then spotted with incremental 10-fold serial dilution onto YPD solid medium and grown for 60 hours at 25 °C, 30 °C, 37 °C, respectively.

## Supplementary information

Supplementary Information.

## Data Availability

The cryoEM structure of MBP-Nop58p_447-R2TP has been deposited to The Electron Microscopy Data Bank with code EMD-20905.
