# Supplementary material for: Yeast R2TP Interacts with Extended Termini of Client Protein Nop58p
Source: Sci Rep. 2019 Dec 27;9:20228. doi: 10.1038/s41598-019-56712-4 (PMC6934851; doi:10.1038/s41598-019-56712-4)
Supplement: Supplementary file 1 — Supplementary Information. [file 41598_2019_56712_MOESM1_ESM.pdf]

Supplementary Materials for

Yeast R2TP Interacts with Extended Termini of Client Protein Nop58p

Ge Yu[1], Yu Zhao[2], Shaoxiong Tian[1], Jay Rai[2], Huan He[2], John Spear[2], Duncan Sousa[3], Jinbo Fan[3], Hong-Guo Yu[3], Scott M. Stagg[1,2], and Hong Li[1,2]*

[1]Department of Chemistry and Biochemistry, Florida State University, Tallahassee, FL 32306, USA.

[2]Institute of Molecular Biophysics, Florida State University, Tallahassee, FL 32306, USA

[3]Department of Biological Science, Florida State University, Tallahassee, FL 32306, USA

Key words: Rvb1;Rvb2;R2TP;AAA+ proteins;Nop58;ribosome biogenesis;cryoEM

Running title: Structure of the Nop58p-bound R2TP complex

*Correspondence to: hong.li@fsu.edu

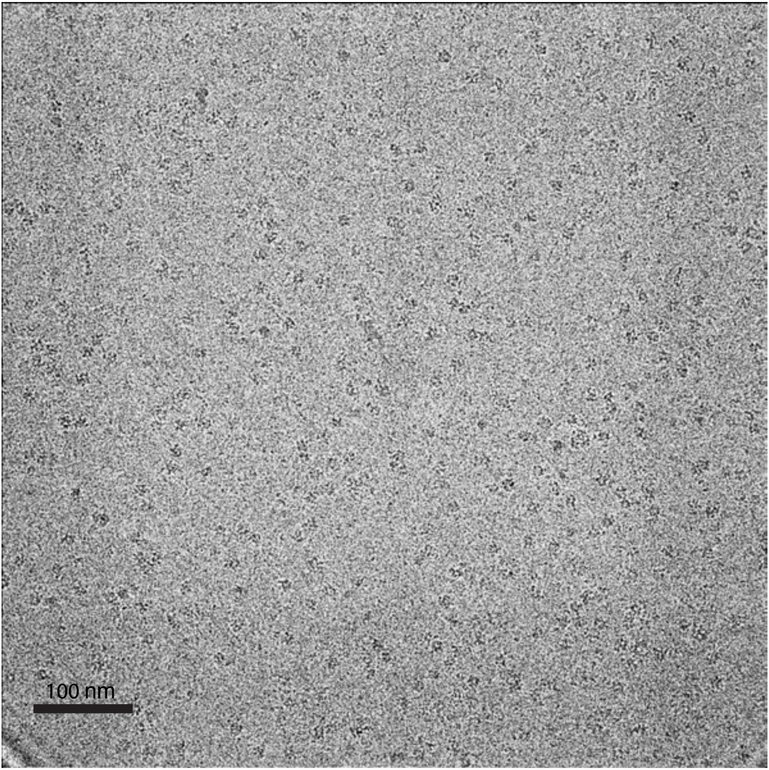

Figure S1

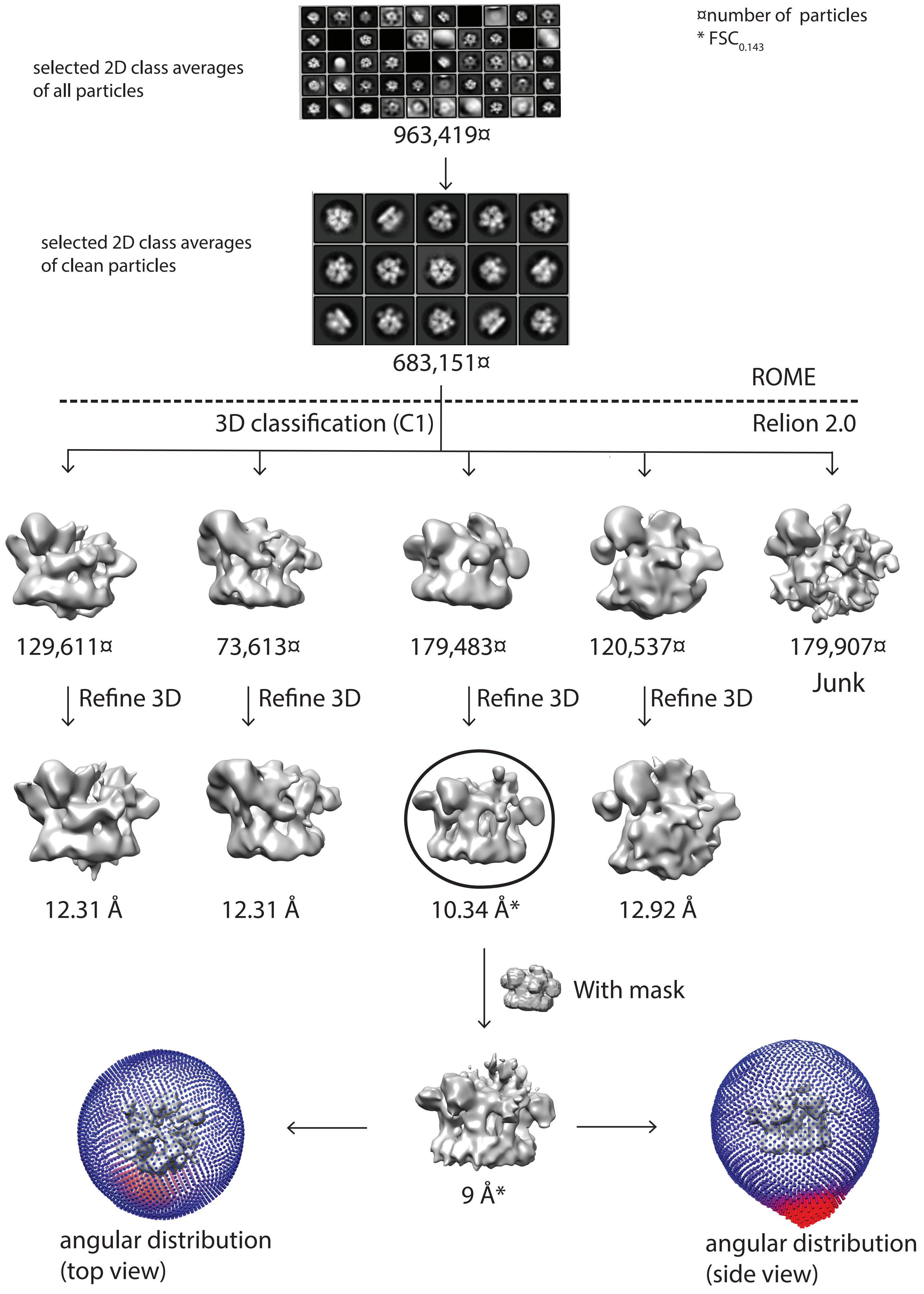
¤number of particles
* FSC$_{0.143}$
selected 2D class averages of all particles
963,419¤
selected 2D class averages of clean particles
683,151¤
ROME
3D classification (C1)
Relion 2.0
129,611¤
73,613¤
179,483¤
120,537¤
179,907¤
Junk
Refine 3D
Refine 3D
Refine 3D
Refine 3D
12.31 Å
12.31 Å
10.34 Å*
12.92 Å
With mask
9 Å*
angular distribution (top view)
angular distribution (side view)

Figure S2

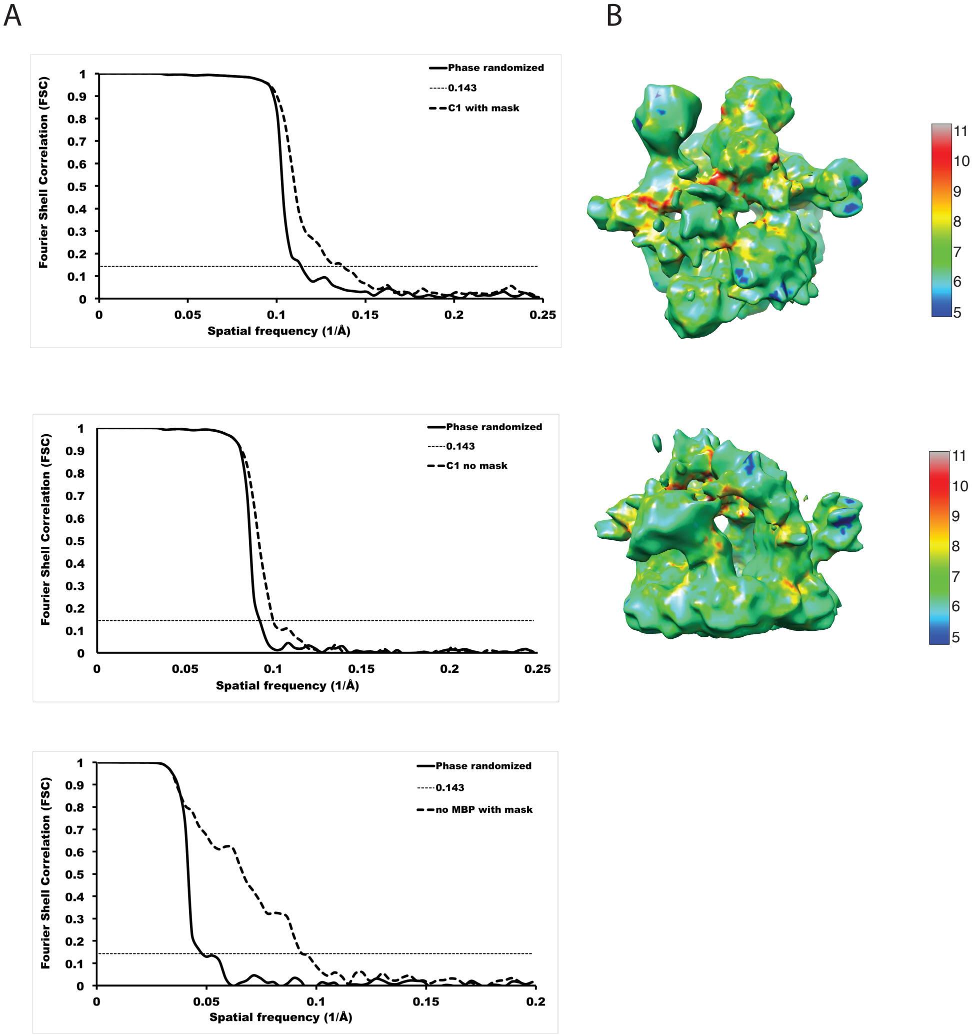
A
B
Phase randomized
0.143
C1 with mask
Fourier Shell Correlation (FSC)
Spatial frequency (1/Å)
Phase randomized
0.143
C1 no mask
Fourier Shell Correlation (FSC)
Spatial frequency (1/Å)
Phase randomized
0.143
no MBP with mask
Fourier Shell Correlation (FSC)
Spatial frequency (1/Å)
11
10
9
8
7
6
5

Figure S3

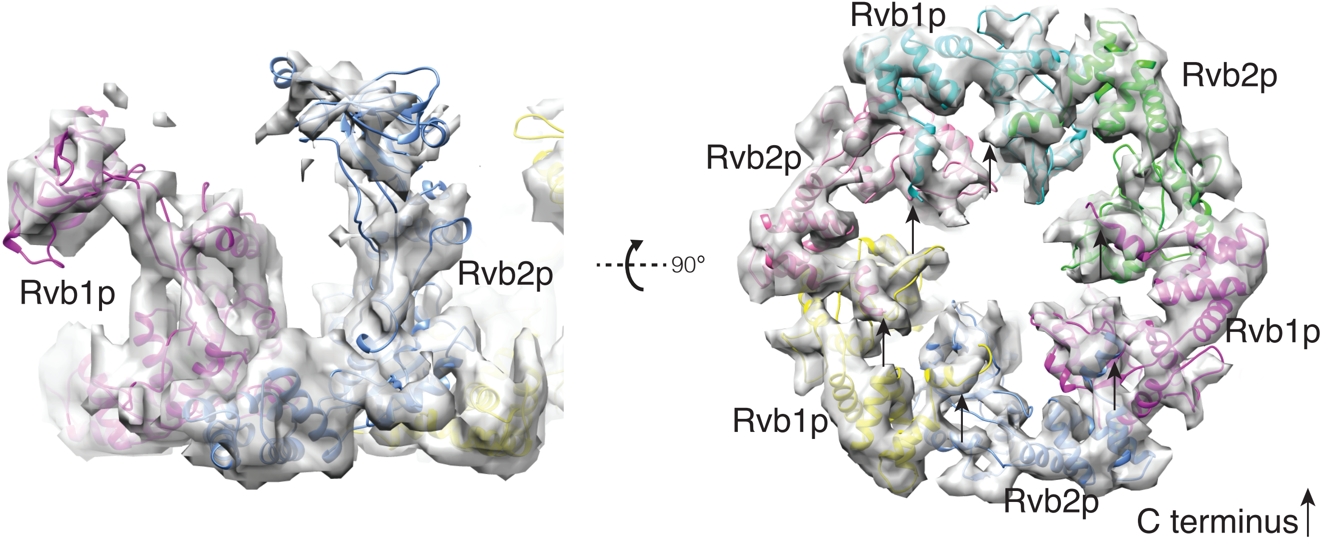
Rvb1p
Rvb2p
90°
Rvb1p
Rvb2p
Rvb2p
Rvb1p
Rvb1p
Rvb2p
C terminus

Figure S4

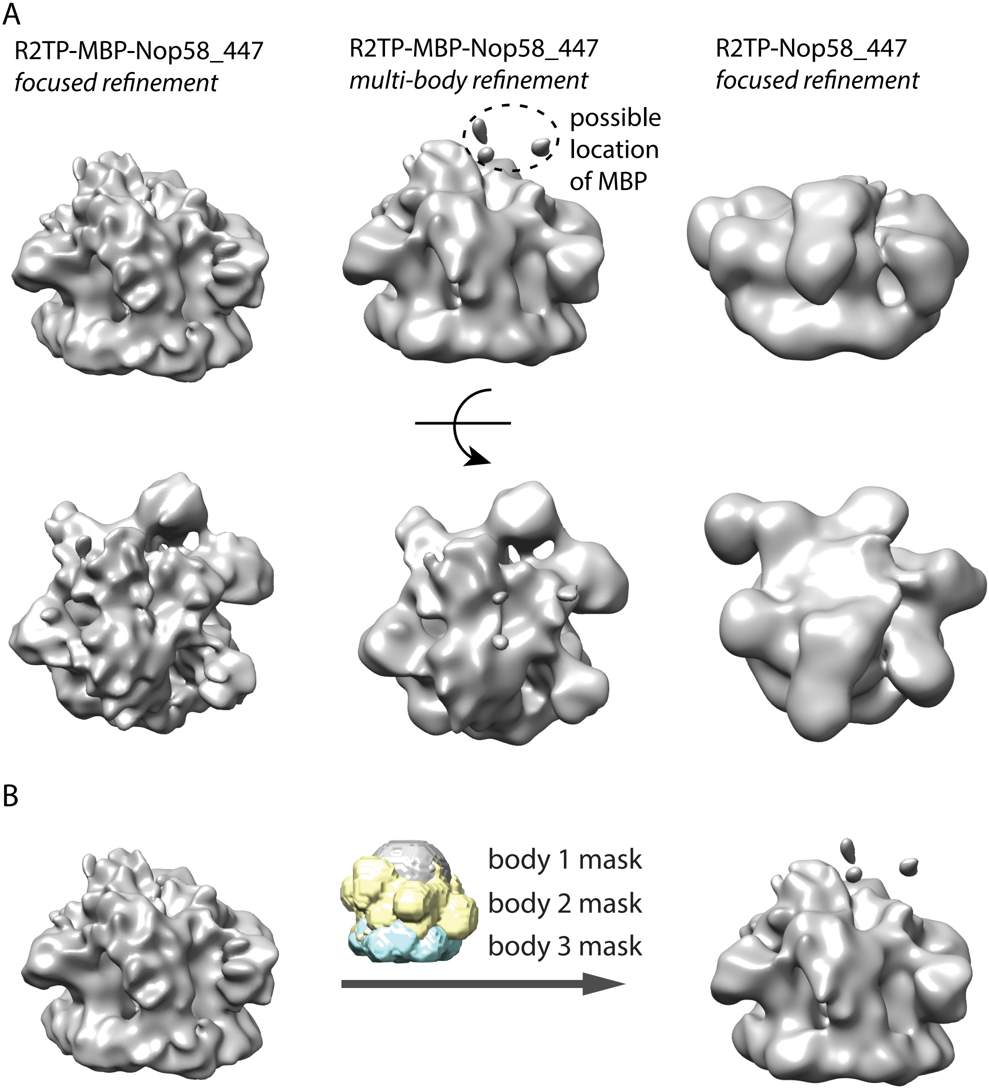
A
R2TP-MBP-Nop58_447
focused refinement
R2TP-MBP-Nop58_447
multi-body refinement
possible
location
of MBP
R2TP-Nop58_447
focused refinement
B
body 1 mask
body 2 mask
body 3 mask

Figure S5

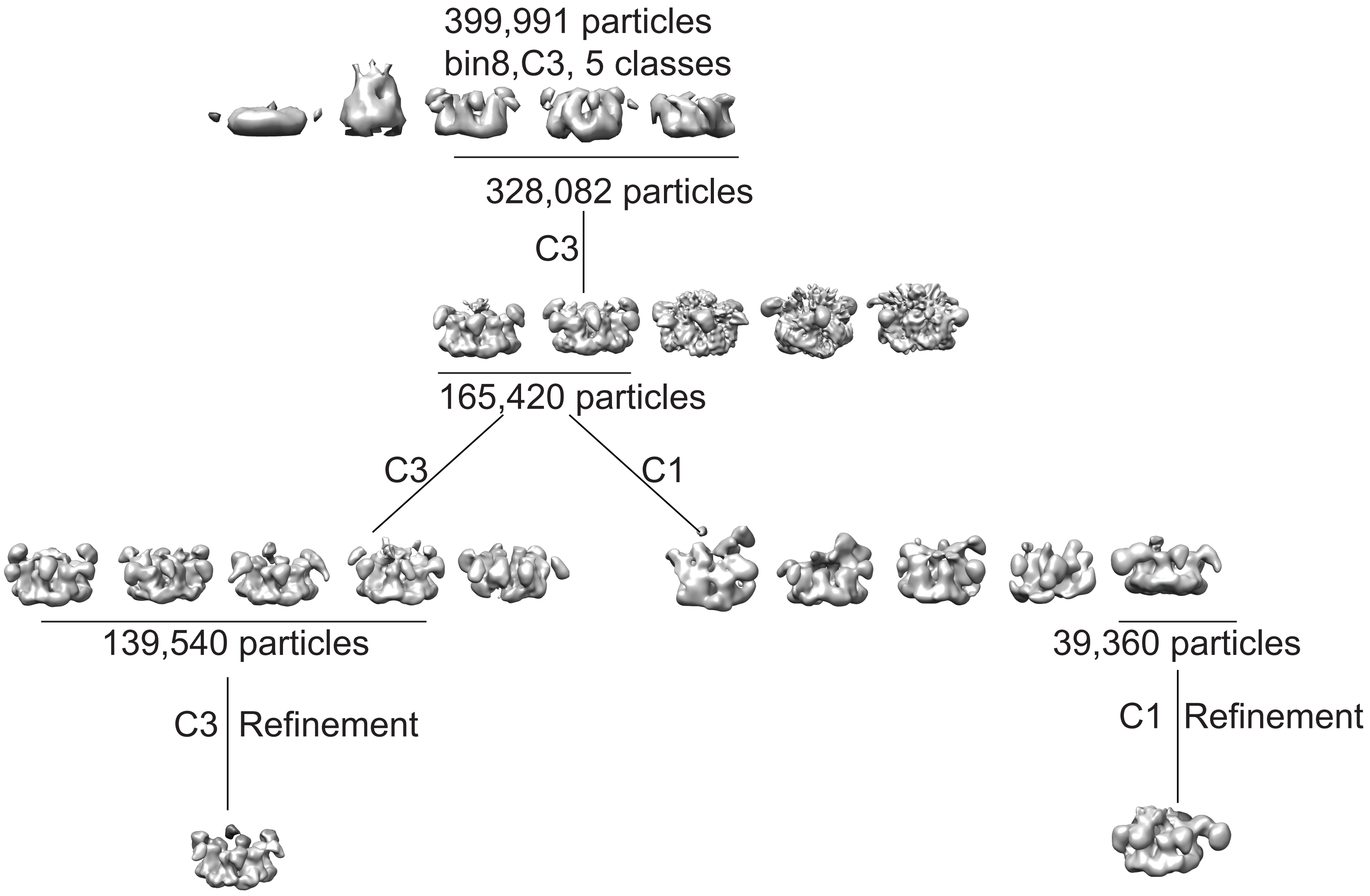
399,991 particles
bin8,C3, 5 classes
328,082 particles
C3
165,420 particles
C3
C1
139,540 particles
39,360 particles
C3
Refinement
C1
Refinement

Figure S6

Table S1. Intra- and Inter-molecular Crosslinks Confirmed by Known Structures*

<table>
<tr><th>Score</th><th>Protein 1</th><th>Site 1</th><th>Protein 2</th><th>Site 2</th><th>Distance (Å)</th></tr>
<tr><td>174</td><td>>sp|P38768|PIH1_YEAST</td><td>K105</td><td>>sp|P38768|PIH1_YEAST</td><td>K160</td><td>18</td></tr>
<tr><td>145</td><td>>sp|P38768|PIH1_YEAST</td><td>K106</td><td>>sp|P38768|PIH1_YEAST</td><td>K164</td><td>13</td></tr>
<tr><td>152</td><td>>sp|P38768|PIH1_YEAST</td><td>K160</td><td>>sp|P38768|PIH1_YEAST</td><td>K275</td><td>14</td></tr>
<tr><td></td><td></td><td></td><td></td><td></td><td></td></tr>
<tr><td>187</td><td>>sp|P25638|TAH1_YEAST</td><td>K79</td><td>>sp|P25638|TAH1_YEAST</td><td>K8</td><td>10</td></tr>
<tr><td>184</td><td>>sp|P25638|TAH1_YEAST</td><td>K79</td><td>>sp|P25638|TAH1_YEAST</td><td>K16</td><td>10</td></tr>
<tr><td></td><td></td><td></td><td></td><td></td><td></td></tr>
<tr><td>124</td><td>>sp|Q12499|NOP58_YEAST</td><td>K84</td><td>>sp|Q12499|NOP58_YEAST</td><td>K393</td><td>67</td></tr>
<tr><td>195</td><td>>sp|Q12499|NOP58_YEAST</td><td>K281</td><td>>sp|Q12499|NOP58_YEAST</td><td>K393</td><td>23</td></tr>
<tr><td>214</td><td>>sp|Q12499|NOP58_YEAST</td><td>K333</td><td>>sp|Q12499|NOP58_YEAST</td><td>K354</td><td>24</td></tr>
<tr><td>209</td><td>>sp|Q12499|NOP58_YEAST</td><td>K358</td><td>>sp|Q12499|NOP58_YEAST</td><td>K366</td><td>16</td></tr>
<tr><td>167</td><td>>sp|Q12499|NOP58_YEAST</td><td>K393</td><td>>sp|Q12499|NOP58_YEAST</td><td>K411</td><td>33</td></tr>
<tr><td>239</td><td>>sp|Q12499|NOP58_YEAST</td><td>K411</td><td>>sp|Q12499|NOP58_YEAST</td><td>K418</td><td>28</td></tr>
<tr><td></td><td></td><td></td><td></td><td></td><td></td></tr>
<tr><td>156</td><td>>sp|Q03940|RUVB1_YEAST</td><td>K290</td><td>>sp|Q12464|RUVB2_YEAST</td><td>S148</td><td>22</td></tr>
<tr><td>159</td><td>>sp|Q03940|RUVB1_YEAST</td><td>K454</td><td>>sp|Q12464|RUVB2_YEAST</td><td>K357</td><td>16</td></tr>
<tr><td>163</td><td>>sp|Q03940|RUVB1_YEAST</td><td>K290</td><td>>sp|Q12464|RUVB2_YEAST</td><td>K157</td><td>38</td></tr>
<tr><td>169</td><td>>sp|Q03940|RUVB1_YEAST</td><td>K134</td><td>>sp|Q12464|RUVB2_YEAST</td><td>K154</td><td>38</td></tr>
<tr><td>171</td><td>>sp|Q03940|RUVB1_YEAST</td><td>K450</td><td>>sp|Q12464|RUVB2_YEAST</td><td>K338</td><td>17</td></tr>
<tr><td>176</td><td>>sp|Q03940|RUVB1_YEAST</td><td>K454</td><td>>sp|Q12464|RUVB2_YEAST</td><td>S339</td><td>16</td></tr>
<tr><td>180</td><td>>sp|Q03940|RUVB1_YEAST</td><td>K290</td><td>>sp|Q12464|RUVB2_YEAST</td><td>K161</td><td>43</td></tr>
<tr><td>180</td><td>>sp|Q03940|RUVB1_YEAST</td><td>K432</td><td>>sp|Q12464|RUVB2_YEAST</td><td>K59</td><td>16</td></tr>
<tr><td>203</td><td>>sp|Q03940|RUVB1_YEAST</td><td>K290</td><td>>sp|Q12464|RUVB2_YEAST</td><td>K154</td><td>19</td></tr>
<tr><td>204</td><td>>sp|Q03940|RUVB1_YEAST</td><td>K454</td><td>>sp|Q12464|RUVB2_YEAST</td><td>K331</td><td>23</td></tr>
<tr><td>260</td><td>>sp|Q03940|RUVB1_YEAST</td><td>K450</td><td>>sp|Q12464|RUVB2_YEAST</td><td>K331</td><td>23</td></tr>
</table>

*The PDB IDs used for confirming intra-molecular crosslinks are: 5WLC (Nop58p), 4CHH (Pih1p N domain), 4CGU (PIh1p C domain and Tah1p). The structures used for confirming inter-molecular crosslinks between Rvb1p and Rvb2p were modeled based on the crystal structure of *Chaetomium thermophilum* Rvb1/Rvb2 (4WVY). Distances between two crosslinked lysine ε-amino groups measured from the structures are listed in the column "Distance", where those in red denote the distances between Rvb1p and Rvb2p DII lysine (or serine) residues.

Table S2. Observed Inter-molecular Crosslinks

Score	Protein 1	Site 1	Protein 2	Site 2
141	>sp|Q03940|RUVB1_YEAST	K274	>sp|Q12499|NOP58_YEAST	K153
141	>sp|Q03940|RUVB1_YEAST	K274	>sp|Q12499|NOP58_YEAST	K311
148	>sp|Q03940|RUVB1_YEAST	K177	>sp|Q12499|NOP58_YEAST	K418
155	>sp|Q03940|RUVB1_YEAST	K277	>sp|Q12499|NOP58_YEAST	K80
155	>sp|Q03940|RUVB1_YEAST	K277	>sp|Q12499|NOP58_YEAST	K311
162	>sp|Q03940|RUVB1_YEAST	K177	>sp|Q12499|NOP58_YEAST	K40
170	>sp|Q03940|RUVB1_YEAST	K277	>sp|Q12499|NOP58_YEAST	K84
174	>sp|Q03940|RUVB1_YEAST	K177	>sp|Q12499|NOP58_YEAST	K24
176	>sp|Q03940|RUVB1_YEAST	K177	>sp|Q12499|NOP58_YEAST	K411
177	>sp|Q03940|RUVB1_YEAST	K283	>sp|Q12499|NOP58_YEAST	K83
187	>sp|Q03940|RUVB1_YEAST	K277	>sp|Q12499|NOP58_YEAST	K153
189	>sp|Q03940|RUVB1_YEAST	K174	>sp|Q12499|NOP58_YEAST	K20
212	>sp|Q03940|RUVB1_YEAST	K174	>sp|Q12499|NOP58_YEAST	K43
222	>sp|Q03940|RUVB1_YEAST	K174	>sp|Q12499|NOP58_YEAST	K40
135	>sp|Q12464|RUVB2_YEAST	N-terminus	>sp|Q12499|NOP58_YEAST	K417
139	>sp|Q12464|RUVB2_YEAST	T366	>sp|Q12499|NOP58_YEAST	K418
152	>sp|Q12464|RUVB2_YEAST	S148	>sp|Q12499|NOP58_YEAST	K197
153	>sp|Q12464|RUVB2_YEAST	K194	>sp|Q12499|NOP58_YEAST	K331
153	>sp|Q12464|RUVB2_YEAST	K198	>sp|Q12499|NOP58_YEAST	K331
154	>sp|Q12464|RUVB2_YEAST	K183	>sp|Q12499|NOP58_YEAST	K411
158	>sp|Q12464|RUVB2_YEAST	K174	>sp|Q12499|NOP58_YEAST	K84
159	>sp|Q12464|RUVB2_YEAST	K276	>sp|Q12499|NOP58_YEAST	K324
165	>sp|Q12464|RUVB2_YEAST	K174	>sp|Q12499|NOP58_YEAST	K411
170	>sp|Q12464|RUVB2_YEAST	K154	>sp|Q12499|NOP58_YEAST	K393
180	>sp|Q12464|RUVB2_YEAST	K157	>sp|Q12499|NOP58_YEAST	K393
130	>sp|P38768|PIH1_YEAST	K275	>sp|Q12499|NOP58_YEAST	K138
141	>sp|P38768|PIH1_YEAST	K275	>sp|Q12499|NOP58_YEAST	K49
142	>sp|P38768|PIH1_YEAST	K41	>sp|Q12499|NOP58_YEAST	K411
151	>sp|P38768|PIH1_YEAST	K160	>sp|Q12499|NOP58_YEAST	K418
198	>sp|P38768|PIH1_YEAST	K162	>sp|Q12499|NOP58_YEAST	K411
199	>sp|P38768|PIH1_YEAST	K233	>sp|Q12499|NOP58_YEAST	K411
216	>sp|P38768|PIH1_YEAST	K160	>sp|Q12499|NOP58_YEAST	K411
142	>sp|Q03940|RUVB1_YEAST	K174	>sp|P38768|PIH1_YEAST	K275
143	>sp|Q03940|RUVB1_YEAST	K177	>sp|P38768|PIH1_YEAST	K248
146	>sp|Q03940|RUVB1_YEAST	K174	>sp|P38768|PIH1_YEAST	K248
163	>sp|Q03940|RUVB1_YEAST	K171	>sp|P38768|PIH1_YEAST	K248
171	>sp|Q03940|RUVB1_YEAST	K177	>sp|P38768|PIH1_YEAST	K239
182	>sp|Q03940|RUVB1_YEAST	K174	>sp|P38768|PIH1_YEAST	K239

145	>sp|Q12464|RUVB2_YEAST	K424	>sp|P38768|PIH1_YEAST	K58
149	>sp|Q12464|RUVB2_YEAST	K157	>sp|P38768|PIH1_YEAST	K10
149	>sp|Q12464|RUVB2_YEAST	K198	>sp|P38768|PIH1_YEAST	K10
150	>sp|Q12464|RUVB2_YEAST	K154	>sp|P38768|PIH1_YEAST	K10
160	>sp|Q12464|RUVB2_YEAST	K194	>sp|P38768|PIH1_YEAST	K275
173	>sp|Q12464|RUVB2_YEAST	K194	>sp|P38768|PIH1_YEAST	K185
191	>sp|Q12464|RUVB2_YEAST	K157	>sp|P38768|PIH1_YEAST	K18
197	>sp|Q12464|RUVB2_YEAST	K183	>sp|P38768|PIH1_YEAST	K275
215	>sp|Q12464|RUVB2_YEAST	K154	>sp|P38768|PIH1_YEAST	K185
